# Fat Composition Measured by Proton Spectroscopy: A Breast Cancer Tumor Marker?

**DOI:** 10.3390/diagnostics11030564

**Published:** 2021-03-21

**Authors:** Almir Bitencourt, Varadan Sevilimedu, Elizabeth A. Morris, Katja Pinker, Sunitha B. Thakur

**Affiliations:** 1 Memorial Sloan Kettering Cancer Center, New York, NY, 10065, USA; almirgvb@yahoo.com.br (A.B.); sevilims@mskcc.org (V.S.); eamorris@ucdavis.edu (E.A.M.); pinkerdk@mskcc.org (K.P.); 2 A.C.Camargo Cancer Center, São Paulo 01509-010, Brazil; 3 School of Medicine, University of California, Davis, CA 95817, USA

**Keywords:** lipids, proton magnetic resonance spectroscopy, breast cancer

## Abstract

Altered metabolism including lipids is an emerging hallmark of breast cancer. The purpose of this study was to investigate if breast cancers exhibit different magnetic resonance spectroscopy (MRS)-based lipid composition than normal fibroglandular tissue (FGT). MRS spectra, using the stimulated echo acquisition mode sequence, were collected with a 3T scanner from patients with suspicious lesions and contralateral normal tissue. Fat peaks at 1.3 + 1.6 ppm (L13 + L16), 2.1 + 2.3 ppm (L21 + L23), 2.8 ppm (L28), 4.1 + 4.3 ppm (L41 + L43), and 5.2 + 5.3 ppm (L52 + L53) were quantified using LCModel software. The saturation index (SI), number of double bods (NBD), mono and polyunsaturated fatty acids (MUFA and PUFA), and mean chain length (MCL) were also computed. Results showed that mean concentrations of all lipid metabolites and PUFA were significantly lower in tumors compared with that of normal FGT (*p* ≤ 0.002 and 0.04, respectively). The measure best separating normal and tumor tissues after adjusting with multivariable analysis was L21 + L23, which yielded an area under the curve of 0.87 (95% CI: 0.75–0.98). Similar results were obtained between HER2 positive versus HER2 negative tumors. Hence, MRS-based lipid measurements may serve as independent variables in a multivariate approach to increase the specificity of breast cancer characterization.

## 1. Introduction

Historically, fat information from magnetic resonance imaging (MRI) examinations is seldom used for diagnostic purposes. In fact, substantial efforts have been invested towards improving fat suppression and producing fat-free images and spectra [1,2,3]. However, in vivo proton MR spectroscopy (MRS) can detect and monitor lipid metabolites in breast tissue [4]. Accumulating evidence points to the dynamic nature of lipid profiles, which can change globally, during the menstrual cycle and carcinogenesis, and locally, during carcinogenesis and gene therapy [5,6,7,8,9].

Altered lipid metabolism is, in fact, an emerging hallmark of breast cancer [10,11]. Overall, approximately 80% of body fat exists in adipocytes, 10% in cell membranes, and another 10% in intracellular lipid droplets. Fat ingested as part of the human diet travels to the small intestine, where it is converted to triacylglycerols; chylomicrons incorporate these triacylglycerols, together with cholesterol and apolipoproteins, and travel with them through the lymphatic system and bloodstream to tissues. There, lipases release the fatty acids, which enter cells, where they are oxidized as fuel or re-esterified for storage (in adipocytes). It is likely that lipid content in adipocytes will reflect a “centralized” distribution which is less sensitive to local changes in the environment compared to lipid content in a tumor. While evidence for several changes in the overall lipid profiles of post-menopausal women with invasive ductal carcinoma (as evidenced by multi-spectral MRI imaging of breast adipose tissue) has recently emerged [6], it is in fact the lipid composition local to the tumor that is more likely to change [9].

Breast cancer cells have been shown to produce and consume lipids, as part of their wide range of tools to survive and sustain proliferative capacity in challenging tumor microenvironments [12]. Unlike most differentiated normal tissues, under nutrient-replete conditions, breast cancer cells synthesize fatty acids de novo [13]. This process meets the demands of rapid metabolism, cell growth, and proliferation: fatty acids are incorporated into new structural lipids including phospholipids and sphingomyelins to meet the demand for rapid growth and cell division common to tumors, and to balance reducing equivalents [10,13,14]. Fatty acid-derived triglycerides and cholesterol esters are stored as lipid droplets, which may provide a rich source of energy and structural building blocks under transient nutrient-depleted conditions [11]. Oncogenic mutations and conditions in the tumor microenvironment such as hypoxia and poor nutrient perfusion have been shown to influence tumor cell capacity for fatty acid chain remodeling by desaturation, which requires molecular oxygen and iron to produce double bonds [15]. This suggests that the fatty acid chain saturation profile of lipids in breast cancer cells may differ from normal glandular tissues, benign lesions, and surrounding adipose tissues. In addition to cell autonomous routes to lipid variation, non-autonomous influences on the lipid composition in surrounding adipose tissue may also occur through pathways that mobilize adipose fat stores to feed tumor progression, or effects stemming from cancer-associated localized inflammation and attendant changes in adipocyte differentiation status [16,17].

The lipid droplets in adipocytes and cancer cells are comprised of triglycerides and cholesterols esters [18]. These lipid droplet fats are mainly oils, and these highly mobile species are good candidates for MRI and spectroscopy. While largely unexplored, the defining characteristics of altered lipid metabolism in and around breast tumors suggest that the fatty acid profile present in cancer may be a diagnostic marker of tumor biology and progression. Therefore, we hypothesized that lipid profiling through MRS imaging may be a powerful tool, helping to discriminate between benign lesions or normal breast tissue and invasive breast cancers. Given the varied genetic backgrounds and metabolic dependencies of different molecular subtypes of breast cancer, this approach may also have the potential to ultimately differentiate between types of invasive cancers.

The purpose of this study was to investigate if breast cancers exhibit different MRS-based lipid composition than normal fibroglandular tissue.

## 2. Materials and Methods

### 2.1. Study Design

Following a protocol approved by our Institutional Review Board (IRB18-213, 25 April 2018), this prospective single-center study included 23 patients with suspicious breast lesions (51.9 ± 10.4-years-old) and the final analysis included 18 patients excluding three patients with benign lesions and two patients due to low spectral quality. All patients gave written informed consent.

### 2.2. In Vivo MRS Acquisitions

**Figure 1:**
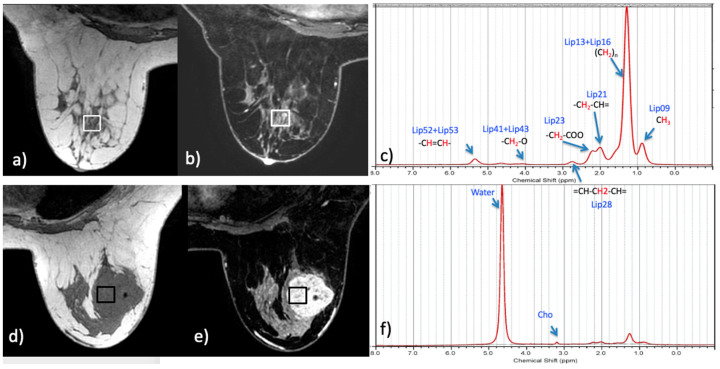
Example of magnetic resonance spectroscopy (MRS) voxel locations (**a**,**d**), T1-weighted fat-unsuppressed image; (**b**,**e**), T1-weighted fat-suppressed contrast-enhanced image and their corresponding experimental spectra (black) overlaid with LCModel fit (red) (**c**,**f**), in normal fibroglandular tissue (**a**–**c**), and tumor tissue in a patient with invasive ductal carcinoma (**d**–**f**).

MRS data were acquired with a 3T MR750 scanner (GE Healthcare, Waukesha, WI, USA), using an 8-channel breast coil. A stimulated echo acquisition mode sequence (TE/TR = 14/1500 ms, 64 averages) was used to acquire fat spectra data from 1 to 2 single voxels (1–2 cc) per patient. Data from two voxels were acquired in patients with suspicious lesions (one over the tumor, and one was placed over glandular tissue in contralateral breast, respectively). Scanning time of each voxel required about 2 min, including shimming. Figure 1 displays examples of voxel locations and experimental spectrum/LCModel fit from a patient tumor and contralateral normal fibroglandular tissue (FGT); efforts were made to keep voxels outside of adipose tissue for all voxels. As previously noted, it was suspected that local alteration of fat profiles in and around the tumor would be more prominent, due to changes in intracellular lipid droplet composition. Adipocytes would likely reflect more of the global fat content; as they are significantly larger (~100 µm) than intracellular lipid droplets’ (~10 µm), their composition would dominate the tissue in which they are included.

### 2.3. Pathology Analysis

Following biopsy, tissues from the lesions probed by MRS were sent for pathology analysis, which included reports of human epidermal growth factor receptor 2 (HER2), estrogen receptor (ER) and progesterone receptor (PR) status for malignant lesions. Three of the biopsied lesions were benign false-positives, resulting in 20 confirmed malignant lesions.

### 2.4. Data Analysis

In vivo datasets were quantified with LCModel version 6.3-1K (http://s-provencher.com/lcmodel.shtml, accessed on 20 March 2021), using SPTYPE = breast. Fat peaks at 1.3 + 1.6 ppm (L13 + L16), 2.1 + 2.3 ppm (L21 + L23), 2.8 ppm (L28), 4.1 + 4.3 ppm (L41 + L43) and 5.2 + 5.3 ppm (L52 + L53) were expressed independently. The saturation index (SI), number of double bods (NDB), mono and polyunsaturated fatty acids (MUFA and PUFA), and mean chain length (MCL) were also computed [19]. R 3.5.2 (R Core Team, 2020) was used for all statistical analysis [20]. Comparison of fat peaks between normal fibroglandular and tumor tissues was performed using the Wilcoxon rank-sum test. For comparison between tumors with different expression of ER, PR, and HER2, fat peaks were adjusted using the Benjamini–Hochberg correction for multiple comparisons [21]. Type I error rate was set at 0.05 (α). Given the small sample size and correlated nature of the various covariates considered, a least absolute shrinkage and selection operator (LASSO) regularization was used to select covariates and perform a multivariable logistic regression analysis, using the *glmnet* functionality in R 3.5.2 [22,23]. The value of the penalty parameter λ was determined using 10-fold cross validation. The discriminative ability of the resulting model was assessed using a receiver operating characteristic (ROC) curve.

**Figure 2:**
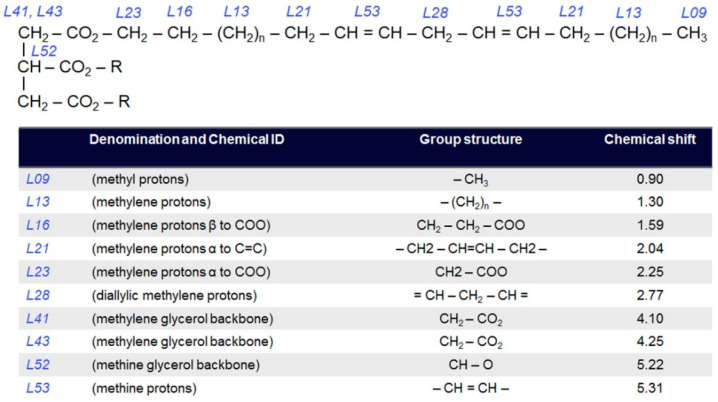
Chemical structure and list with expected resonances in lipids. Adapted with permission from ref. [24]. Copyright © 2011 copyright John Wiley and Sons.

Figure 2, adapted from a previous study [24], displays expected resonances and their position in the fatty acid chain. Only a limited fraction of those can be independently quantified in vivo at 3T; in particular, only five lipid ratios have consistent Cramer–Rao lower bound values below 10%. While all ten fat resonances in Figure 2 were included in the basis set, separately fit, and used for the computation of NDB, SI, MUFA and PUFA, such separation came at the expense of larger measurement variability, translating ultimately into lower normal/cancer separation power.

## 3. Results

**Figure 3:**
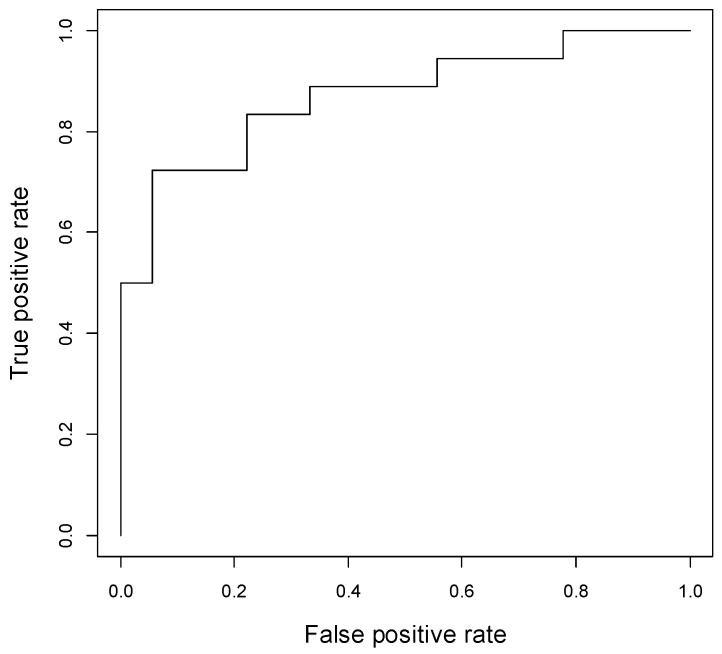
ROC curve of the L21 + L23 lipid peak for differentiating tumor from normal FGT, with an AUC of 0.87 (95% CI: 0.75–0.98).

Table 1 presents a summary of our measures of lipid composition from the study subjects. The mean concentrations of all lipid metabolites and PUFA were significantly lower in tumors compared with that of normal FGT (*p* ≤ 0.002 and 0.04, respectively). Further, multivariable analysis yielded significant difference in L21 + L23 between tumors and normal FGT, with a coefficient of −4.74, which means that as this lipid peak increases in value, the probability of tumor diagnosis decreases. The area under the curve (AUC) was 0.87 (95% CI: 0.75–0.98) (Figure 3). Using an internal validation dataset consisting of randomly selected 30% of the entire dataset, the AUC of the resulting ROC curve was 0.88 (95% CI: 0.63–1.00).

From the 18 tumors, 10 were ER positive, 8 were PR positive and 7 were HER2 positive; two were triple negative, one was triple positive, and the rest had various combinations of ER, PR, and HER2 expression. Table 2 presents the fat peak p-values obtained on univariate analysis for differentiating tumors grouped with respect to ER, PR, and HER2 status. Only the L52 + L53 peak showed statistically significant differences between tumors based on ER and PR status, whereas multiple lipid parameters showed statistically significant differences between tumors based on the HER2 status.

## 4. Discussion

In this work, we have analyzed the fat profile of breast lesions and normal fibroglandular tissue from the contralateral healthy breast in a prospective in vivo MRS study. The results evidenced differences between fat metabolites in the two tissues, mainly characterized by a decrease in the L21 + L23 peak in invasive breast cancers.

Early in vivo MRS studies reported differences in water and fat resonances between normal and tumor tissues, using parameters like water-to-fat ratio [25]. Invasive carcinomas are high in water content with very low levels of lipid when compared to normal fibroglandular tissue. Generally, however, such parameters had limited diagnostic utility due to significant overlap between benign and malignant lesions, and to the variability of water content, which is dependent on breast composition and menstrual cycle [26].

Recently, Agarwal et al. showed that the fat fraction measured by in vivo MRS is significantly lower in malignant breast tissues when compared with benign and normal breast tissues [27]. Prior studies also found that postmenopausal women with breast cancer had lower MUFA and higher saturated fatty acid than those with benign tissue [6,28]. Another previous study based on six types of cancer has shown that activation of de novo lipogenesis is an early and common event in the cancer microenvironment [29]. Multivariate statistical analysis coupled with lipid distribution images revealed that significantly increased levels of MUFA relative to PUFA were observed in the cancer microenvironment compared with the adjacent normal tissue.

In a prior retrospective study that evaluated lipid metabolism by using breast MRS in 168 patients, malignant lesions showed significantly lower L09, L21 + L23, and L52 + L53 values than benign lesions [9]. In this prior study, lipid areas under the peak were normalized with respect to MRS voxel volume prior to statistical analysis. These findings suggest that MRS lipids concentrations could potentially be used to reduce false-positive results in breast MRI.

The current prospective study found that all lipid metabolite concentrations and the PUFA fraction were significantly lower in tumors compared with normal FGT, consistent with previous reports. Multivariable analysis yielded an AUC of 0.87 for the L21 + L23 peak in separating tumor tissues from normal tissue. We used the LASSO algorithm, which is a regularization algorithm that aids in appropriate variable selection while controlling for the variability of parameter estimates. It increases prediction accuracy, which is supported by the findings of our multivariate analysis. Our multivariate analysis indicates that the AUC yielded by the L21 + L23 peak in differentiating tumor tissue from normal tissue was 0.87, which is indicative of a good performance of the peak, thus also making the L21 + L23 peak a definitive entity for identifying cancers.

Despite the small sample sizes, we also observed differences in lipid peaks between tumors grouped with respect to ER, PR, and HER2 status. Although the analysis between receptor types was only exploratory in nature, in the present analysis, only the L52 + L53 peak was found to be lower in ER and PR negative tumors, while multiple lipid parameters were found to be lower in HER2 positive tumors. The prior retrospective study also showed lower lipid metabolite concentrations in luminal cancers compared with non-luminal cancers; however, only the L28 peak remained significantly different between these cancer groups after multiple comparison corrections [9].

The slight differences in results between the prior retrospective and current prospective study may be easily explained by the different experimental parameters. Voxels size, field strength, and other acquisition parameters were different; for example, the echo time of the prior study was longer and was optimized for improved choline detection, whereas the higher signal-to-noise ratio afforded by the higher field strength and the shorter echo time of the current study should have yielded more accurate quantification. It is to be noted, however, that the prior study was better powered than the current one, and the mean voxel size of 4.4 cc of the prior study was also larger than the voxel size of 1–2 cc of the current study. These differences could easily negate the advantages of shorter echo time and higher field strength of the current study. The focus of this study was more on investigating different lipid components for differentiating tumor tissue from normal fibroglandular tissue; more highly powered studies that include more benign lesions will be needed to elucidate the noted differences.

This study has some limitations. First, this is a small dataset and as such, the estimates cannot be generalizable; further research with larger sample sizes should be performed in order to conclusively validate our findings [30]. Second, in vivo MRS was performed in single voxels. Multi-voxel MRS, simultaneously collecting data from planes or 3D volumes, may improve the characterization of lipid metabolism in the breast. In addition, semi-LASER pulse sequences with adiabatic refocusing radiofrequency pulses may afford the collection of 2D or 3D MRS with uniform B1 homogeneity over the MRS volume [31]. We also note that lipid metabolites can be detected with high signal to noise at short TE durations. Additionally, one can apply echo planar spectral imaging techniques for speeding up 2D/3D MRSI acquisition times and making this tool clinically feasible. Finally, in this study, we only investigated the differentiation of normal FGT and breast cancers. We expect that these results will also translate to the differentiation of benign and malignant lesions as altered aberrant lipid metabolism is an emerging hallmark of breast cancer lesions and will be able to add specificity on breast MR evaluation, which is the aim of a future study.

In conclusion, lipid profile acquired on in vivo MRS can help to differentiate malignant from benign tissues. Overall, it is unlikely that fat lipid ratios can serve as sole, independent breast tumor discriminators. They can, however, serve as variables in a multi-parametric approach for better characterization of breast lesions, hopefully leading to increased breast cancer detection specificity. MRS lipid composition data can be acquired through a very short (1–2 min) acquisition and should be completely independent of any other MRI-based measurements (such as permeability evidenced by dynamic contrast-enhanced MRI or tissue cellularity evidenced by diffusion-weighted imaging).

## Figures and Tables

**Table 1:** Fat peaks (concentrations in millimoles) obtained for all voxels, normal fibroglandular tissue (FGT) and tumors. *p*-values refer to the comparison of fat peaks between normal FGT and tumors.

Characteristic Peak	All Voxels, *n* = 36	Normal FGT, *n* = 18	Tumor, *n* = 18	*p*-Value
L09, median (IQR)	0.03 (0.01, 0.10)	0.08 (0.04, 0.12)	0.01 (0.01, 0.03)	<0.001
L13 + L16, median (IQR)	0.23 (0.09, 0.66)	0.49 (0.27, 0.84)	0.09 (0.03, 0.20)	<0.001
L21 + L23, median (IQR)	0.04 (0.02, 0.12)	0.10 (0.05, 0.15)	0.02 (0.01, 0.04)	<0.001
L28, median (IQR)	0.004 (0.002, 0.012)	0.009 (0.004, 0.016)	0.001 (0.000, 0.004)	<0.001
L41 + L43, median (IQR)	0.004 (0.001, 0.012)	0.010 (0.004, 0.014)	0.002 (0.000, 0.004)	<0.001
L52 + L53, median (IQR)	0.007 (0.004, 0.017)	0.012 (0.007, 0.023)	0.005 (0.001, 0.006)	0.002
SI, median (IQR)	9.08 (7.34, 10.93)	9.08 (7.99, 11.23)	9.09 (6.42, 10.82)	0.4
NDB, median (IQR)	0.28 (0.13, 0.41)	0.20 (0.14, 0.35)	0.33 (0.14, 0.45)	0.3
PUFA, median (IQR)	0.16 (0.11, 0.24)	0.23 (0.15, 0.33)	0.14 (0.09, 0.20)	0.04
MUFA, median (IQR)	0.47 (0.33, 0.56)	0.38 (0.30, 0.54)	0.51 (0.45, 0.57)	0.085
MCL, median (IQR)	14.9 (13.3, 17.9)	14.4 (13.7, 17.9)	15.4 (12.2, 17.7)	0.6

**Table 2:** Fat peak *p*-values obtained for differentiating tumors grouped with respect to ER, PR, and HER2 status.

Characteristic Peak	*p*-Value		
	ER+ (*n* = 5) vs. ER− (*n* = 13)	PR+ (*n* = 3) vs. PR− (*n* = 15)	HER2+ (*n* = 8) vs. HER2− (*n* = 10)
L09	0.24	0.22	0.07
L13 + L16	0.24	0.22	0.06
L21 + L23	0.22	0.22	0.06
L28	0.22	0.22	0.04
L41 + L43	0.22	0.22	0.04
L52 + L53	0.03	0.03	0.03
SI	0.24	0.22	0.14
NDB	0.22	0.22	0.11
PUFA	0.22	0.22	0.04
MUFA	0.90	0.90	0.90
MCL	0.66	0.22	0.11

## Data Availability

Data will be made available upon reasonable request.
